# Exosomal miR-126 blocks the development of non-small cell lung cancer through the inhibition of ITGA6

**DOI:** 10.1186/s12935-020-01653-6

**Published:** 2020-12-15

**Authors:** Mingjun Li, Qianqian Wang, Xiaofei Zhang, Ningning Yan, Xingya Li

**Affiliations:** Department of Oncology, The First Affiliated Hospital of Zhengzhou University, No. 1 East Jianshe Road, Zhengzhou, 450052 Henan China

**Keywords:** Exosomes, miR-126, ITGA6, NSCLC

## Abstract

**Background:**

Exosomes, emerging mediators of intercellular communication, are reported to transfer certain non-coding RNAs, such as microRNAs (miRNAs), which play a crucial role in cancer progression. The objective of this study was to determine the function of exosomal miR-126 and provide a novel mechanism of miR-126 action in NSCLC.

**Methods:**

The morphology of exosomes was identified by transmission electron microscope (TEM), and the exosomal surface markers were quantified by western blot. The expression of miR-126 and integrin alpha-6 (ITGA6) mRNA was measured by quantitative real-time polymerase chain reaction (qRT-PCR), and ITGA6 protein expression was determined by western blot. For functional analyses, cell proliferation was assessed by colony formation assay and MTT assay. Cell cycle and cell apoptosis were monitored using flow cytometry assay. Cell migration and invasion were determined by transwell assay. ITGA6 was predicted as a target of miR-126 by bioinformatics analysis, which was verified by dual-luciferase reporter assay. The role of exosomal miR-126 in vivo was determined by Xenograft tumor models.

**Results:**

NSCLC serum-derived exosomes harbored low expression of miR-126 and promoted NSCLC cell proliferation, cell cycle progression, cell migration and invasion. NSCLC serum-derived exosomes loaded with miR-126 mimic inhibits NSCLC cell proliferation, colony formation, migration and invasion but induced cell cycle arrest and apoptosis. Besides, exosomal miR-126 also blocked tumor growth in vivo. In mechanism, ITGA6 was a target of miR-126, and exosomal miR-126 weakened these NSCLC cell malignant behaviors and inhibited tumor growth by degrading the expression of ITGA6.

**Conclusion:**

Exosomal miR-126 blocked the progression of NSCLC through the mediation of its target gene ITGA6, and exosomal miR-126 might be used as a promising biomarker for NSCLC therapy.

## Introduction

Lung cancer is one of the most deadly diseases in the world. In 2019, there were estimatedly more than 228,150 new diagnoses and 142,670 deaths in the United States [1]. Non-small cell lung cancer (NSCLC) accounts for about 85% of lung cancer cases and is mainly composed of squamous cell carcinoma (SCC) and adenocarcinoma [2]. Although diagnosis and existing molecular therapies have made progress, the 5-year overall survival rate of NSCLC patients is still poor, only 19% [1, 3]. The main reason for the poor prognosis is that most patients are diagnosed at the advanced stage due to the lack of specific biomarkers and early detection tools.

Exosomes are small extracellular vesicles, about 30–150 nm in diameter, with unique characteristics, including immunocompatibility and low toxicity [4, 5]. Several proteins have been identified on the surface of exosomes, such as tetraspanins (CD63 and CD81), heat shock protein (HSP70), lysosomal protein (Lamp2b), etc. [6]. Exosomes generally transfer biomolecules, such as proteins, lipids, mRNA and microRNA (miRNA), between different cells, thus playing functions in different pathophysiological conditions [7, 8]. Tumor-derived exosomes interact with other cells in the tumor microenvironment to regulate tumor progression, metastasis, and immune escape [9]. Although extracellular miRNAs can be combined with certain RNA-binding proteins and transported in the circulatory system, exosome miRNAs are more stable and are ideal targets for cancer treatment [10].

MiRNAs are endogenous small non-coding RNAs, with 19–22 nucleotides in length [11]. In some cases, miRNAs can bind to specific regions of target mRNA, such as 3′ untranslated region (3′UTR), to guide degradation or translation inhibition of target mRNA [11]. Accordingly, miRNAs play key functions in various biological processes, including tumorigenesis and development, cell proliferation, migration, invasion and apoptosis, by targeting target mRNAs [12, 13]. According to reports, the secretion of exosomes may lead to changes in the behavior of cancer cells [14], and a variety of tissue-specific miRNAs and circulating miRNAs with high sensitivity and specificity can be used as potential biomarkers for diagnosis and treatment of NSCLC patients [15, 16]. For example, serum-derived exosomal miR-451a showed high expression in the NSCLC patients, and exosomal miR-451a was significantly associated with lymph node metastasis, vascular invasion, and stage [17]. A previous study held the view that exosomal miR-126 was an ideal biomarker in NSCLC diagnosis and treatment because exosome-enriched miR-126 inhibited NSCLC cell growth and induced loss of malignant behaviors [18]. However, the role of exosomal miR-126 is not fully determined, and the related mechanism of miR-126 action is not fully understood.

In the present study, we investigated the expression of miR-126 in NSCLC serum-derived exosomes and cell lines. Besides, the function of exosomal miR-126 on cell cycle, proliferation, migration, invasion and apoptosis was assessed. Moreover, we identified the target mRNAs of miR-126 and thus provided a novel mechanism of exosomal miR-126 associated with integrin alpha-6 (ITGA6) in NSCLC, aiming to supply an ideal biomarker for NSCLC diagnosis and treatment.

## Materials and methods

### Serum samples

NSCLC patients (n = 20) and healthy controls (n = 20) were recruited from the First Affiliated Hospital of Zhengzhou University. After obtaining written informed consent from these subjects, blood samples were collected to obtain serum samples by centrifugation. The serum samples in cryopreservation tubes were treated with liquid nitrogen and stored at − 80 °C conditions. This study design obtained the approval of the Ethics Committee of the First Affiliated Hospital of Zhengzhou University.

### Exosome isolation and transmission electron microscope (TEM) identification

Serum samples were treated with ExoQuick exosome precipitation solution (System Biosciences, Mountain View, CA, USA) and then subjected to differential centrifugation to obtain exosomes, following the manuscript’s guidelines.

For the TEM morphology identification, the exosome preparation was fixed with 4% paraformaldehyde for 1 h and washed with PBS. Then, the exosomes pellet was fixed in 2.5% glutaraldehyde and loaded on formvar carbon-coated electron microscopy grids, maintaining for 5 min at room temperature. Afterwards, standard 1% uranyl acetate (pH 4.0) was utilized for exosome pellet staining for 10 min at room temperature. The grid was washed with PBS and subsequently observed under a TEM (H7500; Hitachi, Tokyo, Japan).

### Western blot

Some markers were quantified by western blot. In brief, total proteins were extracted using RIPA Lysis and Extraction Buffer or Exosome Protein Extraction Kit (EZBioscience, Roseville, CA, USA) according to the protocols. Then, proteins were isolated by 10% SDS-PAGE and electro-transferred on PVDF membranes (Bio-Rad, Hercules, CA, USA). Subsequently, the membranes containing proteins were seriatim incubated with the primary and secondary antibodies for indicated time, including anti-heat shock protein 70 (anti-HSP70; ab47454; Abcam, Cambridge, MA, USA), anti-CD63 (ab134045; Abcam), anti-CD81 (ab109201; Abcam), anti-E-cadherin (ab40772; Abcam), anti-N-cadherin (ab76011; Abcam), anti-Vimentin (ab8069; Abcam), anti-ITGA6 (ab235905; Abcam) and anti-GAPDH (ab8245; Abcam), and Goat Anti-Mouse/Rabbit (ab205719 and ab205718; Abcam). The protein signals were visualized using the ECL Western Blotting Substrate (Bio-Rad) and quantified using Image J software (NIH, Bethesda, MA, USA).

### Cell lines

Human bronchial epithelioid cells (HBE) and NSCLC cells (A549 and H460) were purchased from Procell (Wuhan, China). According to the instruction, HBE and H460 cells were cultured in RPMI1640 (Procell) supplemented with 10% exosomes-free FBS, and A549 cells were cultured in Ham’s F-12 K (Procell) supplemented with 10% exosomes-free FBS at 37 °C incubators containing 5% CO_2_.

### Cell co-culture

A549 and H460 cells in matched culture medium containing 10% exosomes-free FBS were seeded into a 24-well plate at a density of 2 × 10^3^ cells/well. At 50–60% confluence, cells were co-incubated with serum-derived exosomes (50 μL/well) for 24 h. Then, cells were collected for functional assays.

### Flow cytometry assay

Cell cycle progression was investigated using the Cell Cycle Analysis Kit (Beyotime, Shanghai, China). Briefly, treated cells were collected, treated with trypsin and then suspended in cooled PBS. Next, cells were fixed with 70% ethanol at 4 °C overnight and then washed with PBS. Finally, cells were stained with propidium iodide (PI) (containing RNase A) at 37 °C for 30 min in a room without light. The cell distribution in different stages was analyzed by a FACScan flow cytometer (BD Bioscience; San Jose, CA, USA).

Cell apoptosis was investigated using the Annexin V-FITC Apoptosis Detection Kit (Beyotime). In brief, cells were collected, treated with trypsin and suspended in PBS. A total of 5 × 10^4^ cells were gathered and treated with 195 μL Annexin V-FITC binding buffer and then stained with 5 μL Annexin V-FITC and 10 μL PI, followed by incubation at room temperature in the dark for 20 min. The apoptotic cells were monitored by flow cytometry assay using a FACScan flow cytometer (BD Bioscience).

### Colony formation assay

A549 and H460 cells were seeded into 6-well plates (200 cells/well) and placed in a 37 °C incubator containing 5% CO_2_ for 2 weeks until forming visible cloned cells. The colonies were washed by PBS, fixed with 4% formaldehyde (Beyotime) and stained with crystal violet (Beyotime). The number of colonies was counted under a microscope (Nikon, Tokyo, Japan).

### MTT assay

A549 and H460 cells were plated into a 96-well plate at the density of 5 × 10^3^ cells/well and cultured in an incubator at 37 °C supplemented with 5% CO_2_. After culturing for 0, 24, 48 and 72 h, 10 μL MTT solution (Beyotime) was added into each well, incubating for another 2 h. Then, DMSO was added to remove formazan. The value of optical density (OD) in each well at 570 nm was measured using a microplate reader (Thermo Fisher Scientific; Waltham, MA, USA).

### Transwell assay

24-well transwell chambers (Corning Incorporated; Corning, NY, USA) pre-coated with or without Matrigel (Corning Incorporated) were used for invasion or migration analysis, respectively. A549 and H460 cells (2 × 10^4^) in serum-depleted medium were seeded into the upper chambers pre-coated with or without Matrigel, and the lower chambers were filled with matched culture medium containing 10% FBS. After 24-h incubation, cells migrated or invaded to the lower surface of chambers were fixed with formaldehyde (Beyotime) and stained with crystal violet (Beyotime). The morphology of migrated or invaded cells was observed using an inverted microscopy (100 × ; Nikon) in at least five fields.

### Quantitative real-time polymerase chain reaction (qRT-PCR)

Total RNA was isolated from cells or exosomes using the Trizol reagent (Beyotime) or Exosomal RNA Isolation Kit (Norgen Biotek, Thorold, Canada), respectively. The quality of the RNA was determined by NanoDrop 2000 (Thermo Fisher Scientific). Then, cDNA was transcribed from 1 μg of total RNA using iScript™ cDNA synthesis kit (Bio-Rad) and amplified for qRT-PCR using SYBR-Green buffer (Bio-Rad) for mRNA. The expression of miRNA was determined using the TaqMan miRNA Assay kit (Applied Biosystems, Foster City, CA, USA) based on the guidelines. The relative expression was calculated using the 2^–ΔΔCt^ method with GAPDH or U6 as the internal reference. The primers sequences were exhibited as follows: miR-126, F 5′-TGTGGCTGTTAGGCATGG-3′ and R 5′-AAGACTCAGGCCCAGGC-3′; U6, F 5′-CTCGCTTCGGCAGCACATATACTA-3′ and R 5′-ACGAATTTGCGTGTCATCCTTGC-3′; ITGA6, F 5′-CACATCTCCTCCCTGAGCAC-3′ and R 5′-TATCTTGCCACCCATCCTTG-3′; GAPDH, F 5′-TGACCACAGTCCATGCCATCAC-3′ and R 5′-GCCTGCTTCACCACCTTCTTGA-3’.

### Transfection in exosomes

MiR-126 mimic (miR-126), miR-126 inhibitor (anti-miR-126) and their matched negative control (miR-NC and anti-miR-NC) were obtained from Ribobio (Guangzhou, China). Overexpression vector pcDNA-ITGA6 (ITGA6) and empty vector (pcDNA; control) were constructed by Sangon Biotech (Shanghai, China). The mimic, inhibitor or plasmid was loaded into exosomes through chemical mediation using the Exo-Fect™ Exosome Transfection Kit (System Biosciences) following the instruction. The efficiency of transfection was examined according to expression level using qRT-PCR or western blot.

### Dual-luciferase reporter assay

ITGA6 was predicted as a putative target of miR-126 by the bioinformatics website (miRDB; https://www.mirdb.org/).

The wild-type sequence and mutant-type sequence (mutation at miR-126 binding site) of ITGA6 3′UTR fragment were generated and amplified to construct pmir-GlO-ITGA6 3′UTR wild-type and pmir-GlO-ITGA6 3′UTR mutant-type recombinant reporter vectors, naming as ITGA6-WT and ITGA6-MUT. A549 and H460 cells were seeded in a 24-well culture plate and transfected with miR-126 or miR-NC and the recombinant vector (ITGA6-WT or ITGA6-MUT), culturing at 37℃ conditions containing 5% CO_2_ for 48 h. Then, cells were collected to measure the relative luciferase activity using the dual-luciferase assay system (Promega, Madison, WI, USA).

### RNA immunoprecipitation (RIP) assay

RIP assay was performed using the Magna RIP™ RNA-Binding Protein Immunoprecipitation Kit (Millipore, Burlington, MA, USA) as described in a previous study [19]. In brief, A549 and H460 cells were lysed using RIP lysis buffer, and cell lysates were incubated with respective antibodies (anti-Ago2 and anti-IgG) coupled to Dynabeads. After the digestion with proteinase K, co-precipitated RNAs were eluted and used for qRT-PCR.

### Xenograft tumor models

Nude mice (BALB/c, female, 6 weeks, 18–22 g) purchased from Shanghai SLAC Animal Center (Shanghai, China) were housed at a constant temperature condition at 25–27 °C, with a humidity of 45–50%. The mice were divided into two groups (n = 5 per group) and subcutaneously injected with A549 cells (2 × 10^6^). Meanwhile, exosomes (30 µg) containing miR-126 or miR-NC (EXO-miR-126 or EXO-miR-NC) were intravenously injected into nude mice through the tail vein every 3 days. One week later, tumor volume (length × width^2^ × 0.5) was recorded every 5 days. After 32 days, all mice were killed to excise tumor tissues for further experiments, including weighting analysis and expression analysis. The animal procedures obtained the permission of the Animal Care and Use Committee of the First Affiliated Hospital of Zhengzhou University.

### Immunohistochemistry (IHC) assay

Simply put, paraffin section of tumor tissues from mice were dewaxed and rehydrated. After antigen retrieval, tissue sections were quenched in 0.3% hydrogen peroxide and blocked using 5% goat serum. The slides were incubated with anti-Ki67 (ab92742; Abcam) or anti-Cleaved-caspase3 (ab2302; Abcam) at 4 °C overnight and then probed with HRP-conjugated secondary antibody (ab205718; Abcam) at room temperature for 1 h. Afterwards, the slides were stained using diaminobenzidine (DAB). The presentation of dark brown was considered to be positive.

### Statistical analysis

The data were analyzed using GraphPad Prism software (v5.0; GraphPad Prism, La Jolla, CA, USA) and shown as mean ± standard deviation. Data comparison was conducted by using Student’s *t* test or analyses of variance (ANOVA) with Tukey post hoc test. *P* value less than 0.05 was regarded as statistically significant. All experiments were repeated at least in triplicate.

## Result

### The identification of exosomes isolated from serum of NSCLC patients

**Fig. 1:**
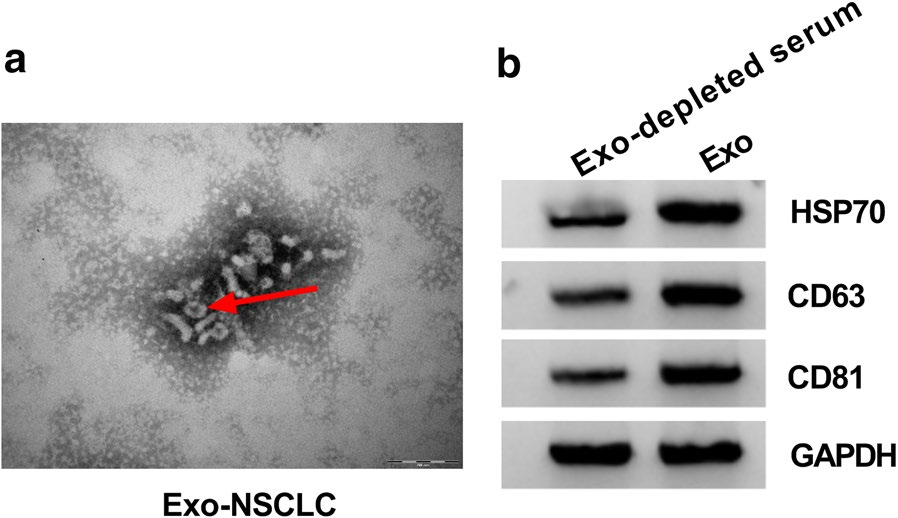
The identification of serum-derived exosomes. **a** The morphology of isolated exosomes was observed by TEM. **b** The expression of exosome surface marker proteins, including HSP70, CD63 and CD81, was detected by western blot. *P < 0.05

Exosomes were isolated from serum samples from NSCLC patients, and the morphology of exosomes was observed under TEM. The image showed that the typical morphology of exosomes was characterized by lipid bilayer membrane-encapsulated nanoparticles (100,000 ×) (Fig. 1a). Besides, the expression of exosomal surface proteins was detected to further verify the existence of exosomes. The data presented that the expression of HSP70, CD63 and CD81 was remarkably increased in NSCLC serum-derived exosomes (Fig. 1b). These suggested that exosomes were successfully isolated from serum samples from NSCLC patients.

### NSCLC serum-derived exosomes promoted cell cycle progression, colony formation, cell migration and invasion but suppressed cell apoptosis in A549 and H460 cells

**Fig. 2:**
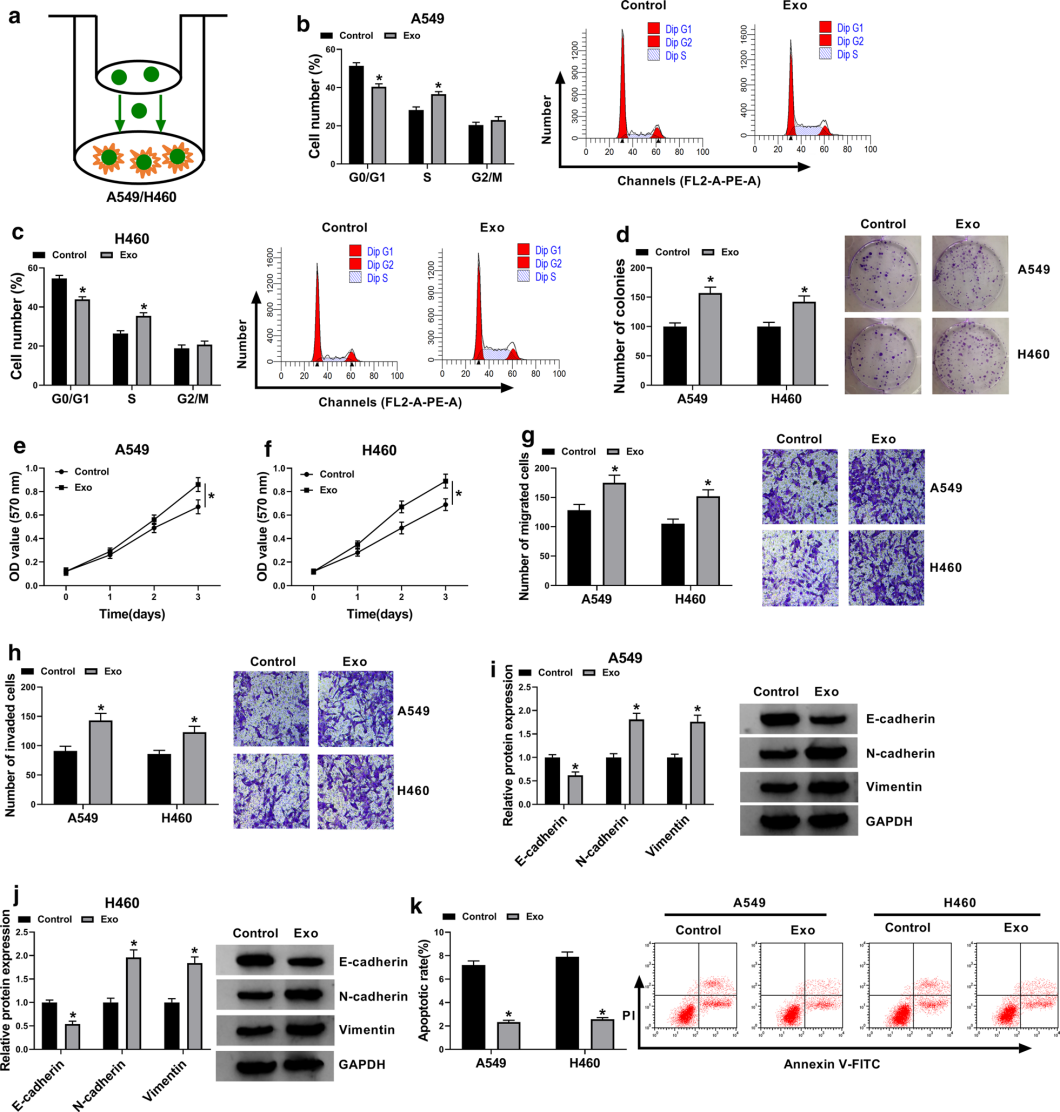
NSCLC serum-derived exosomes promoted NSCLC cell malignant behaviors in vitro. **a** A549 and H460 cells were co-cultured with NSCLC serum-derived exosomes, with exosomes-depleted serum as the control. In these co-cultured cells, **b**, **c** cell cycle was detected by flow cytometry. **d** The ability of colony formation was assessed by colony formation assay. **e**, **f** Cell proliferation was assessed by MTT assay. **g**, **h** Cell migration and cell invasion were investigated using transwell assay. **i**, **j** The expression of E-cadherin, N-cadherin and Vimentin was determined by western blot. **k** Cell apoptosis was monitored by flow cytometry assay. *P < 0.05

To study the effects of NSCLC serum-derived exosomes on biological functions of NSCLC cells in vitro, we exposed A549 and H460 cells to NSCLC serum-derived exosomes (Fig. 2a). In function, NSCLC serum-derived exosomes promoted cell cycle progression compared to Control (Fig. 2b, c). Besides, NSCLC serum-derived exosomes strengthened the ability of colony formation compared to Control (Fig. 2d). MTT assay was performed to determine cell proliferation, and the data showed that serum exosomes reinforced cell proliferation (Fig. 2e, f). As expected, the capacities of cell migration and cell invasion were also promoted by the addition of NSCLC serum-derived exosomes (Fig. 2g, h). E-cadherin, N-cadherin and Vimentin are classical markers of epithelial-to-mesenchymal transition (EMT) that play a vital role in invasion and metastasis of carcinomas [20]. The expression levels of E-cadherin, N-cadherin and Vimentin proteins were investigated here to monitor the ability of migration and invasion. The data showed that the expression of E-cadherin was impaired, while the expression of N-cadherin and Vimentin was elevated in cells containing NSCLC serum-derived exosomes (Fig. 2i, j), suggesting that NSCLC serum-derived exosomes induced A549 and H460 cell migration and invasion. Moreover, NSCLC serum-derived exosomes weakened the number of apoptotic cells (Fig. 2k). The data suggested that NSCLC serum-derived exosomes induced NSCLC cell malignant phenotypes.

### Exosomal miR-126 suppressed NSCLC development in vitro

**Fig. 3:**
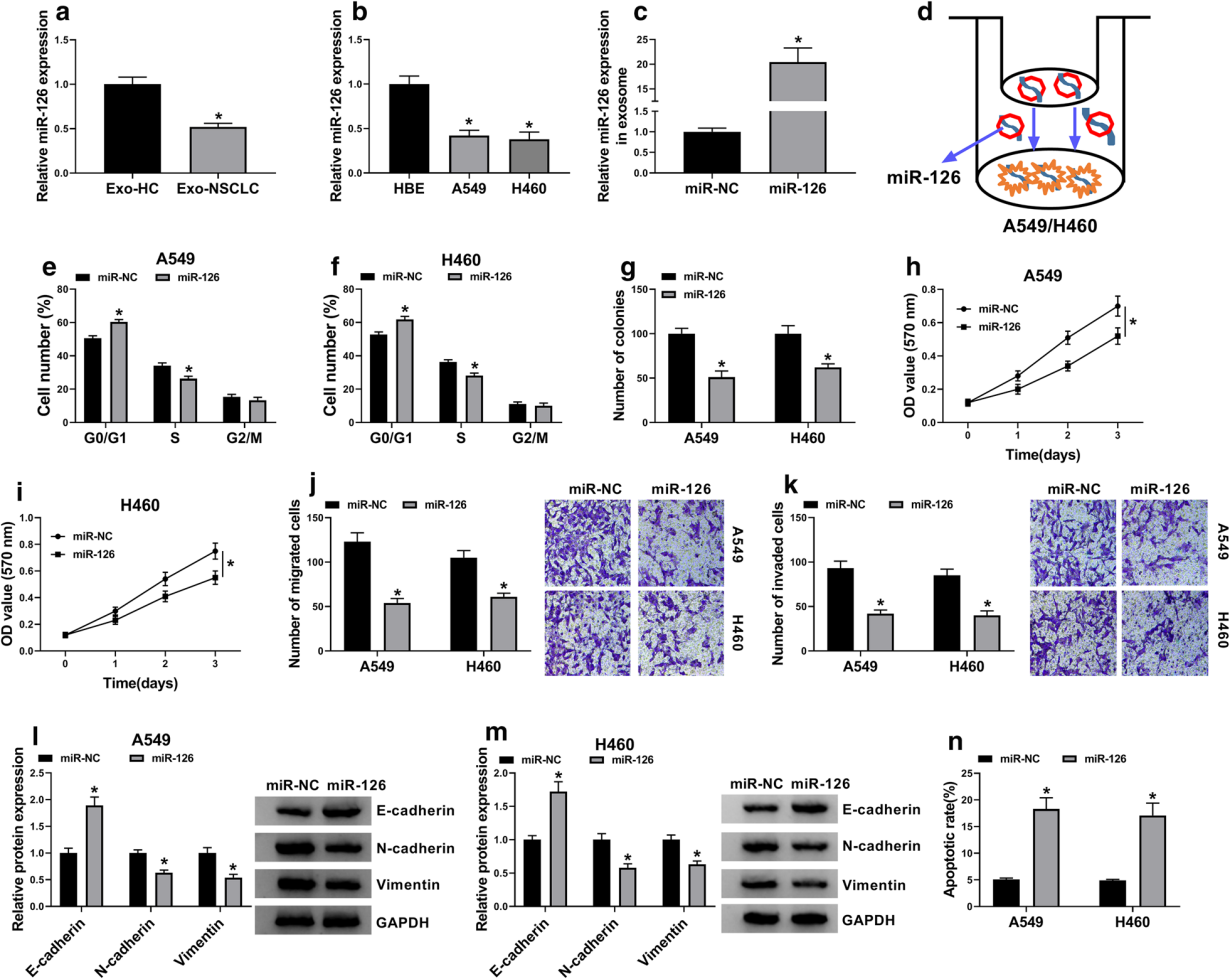
Exosomal miR-126 suppressed NSCLC cell malignant behaviors in vitro. **a** The expression of miR-126 in serum-derived exosomes from NSCLC patients and healthy controls was detected by qRT-PCR. **b** The expression of miR-126 in A549, H460 and HBE cells was measured by qRT-PCR. **c** The efficiency of miR-126 mimic in exosomes was examined using qRT-PCR. **d** A549 and H460 cells were co-cultured with NSCLC serum-derived exosomes transfected with miR-126 or miR-NC. In these cells, **e**, **f** cell cycle was investigated using flow cytometry assay. **g** The ability of colony formation was assessed by colony formation assay. **h**, **i** Cell proliferation was monitored by MTT assay. **j**, **k** Cell migration and cell invasion were detected using transwell assay. **l**, **m** The expression of E-cadherin, N-cadherin and Vimentin was determined by western blot. **n** Cell apoptosis was evaluated by flow cytometry assay. *P < 0.05

We noticed that the expression of miR-126 was lower in serum-derived exosomes from NSCLC patients than that from healthy controls (Fig. 3a). Besides, the expression of miR-126 was also decreased in A549 and H460 cells compared with that in HBE cells (Fig. 3b). Then, NSCLC serum-derived exosomes were transfected with miR-126 or miR-NC. The expression of miR-126 was significantly enhanced in exosomes transfected with miR-126 compared to miR-NC (Fig. 3c). Subsequently, A549 and H460 cells were co-cultured with these transfected exosomes (Fig. 3d). In function, exosomal miR-126 significantly induced cell cycle arrest (Fig. 3e, f). In addition, the abilities of colony formation, cell proliferation, cell migration and invasion were all blocked by the treatment of exosomal miR-126 (Fig. 3g, k). Additionally, the expression of E-cadherin was enhanced, while the expression of N-cadherin and Vimentin was impaired in A549 and H460 treated with exosomal miR-126 compared to miR-NC (Fig. 3l, m). Exosomal miR-126 markedly induced A549 and H460 cell apoptosis (Fig. 3n). To conclude, exosomal miR-126 contributed to the inhibition of NSCLC cell malignant phenotypes.

### MiR-126 bound to ITGA6 3′UTR and suppressed ITGA6 expression

**Fig. 4:**
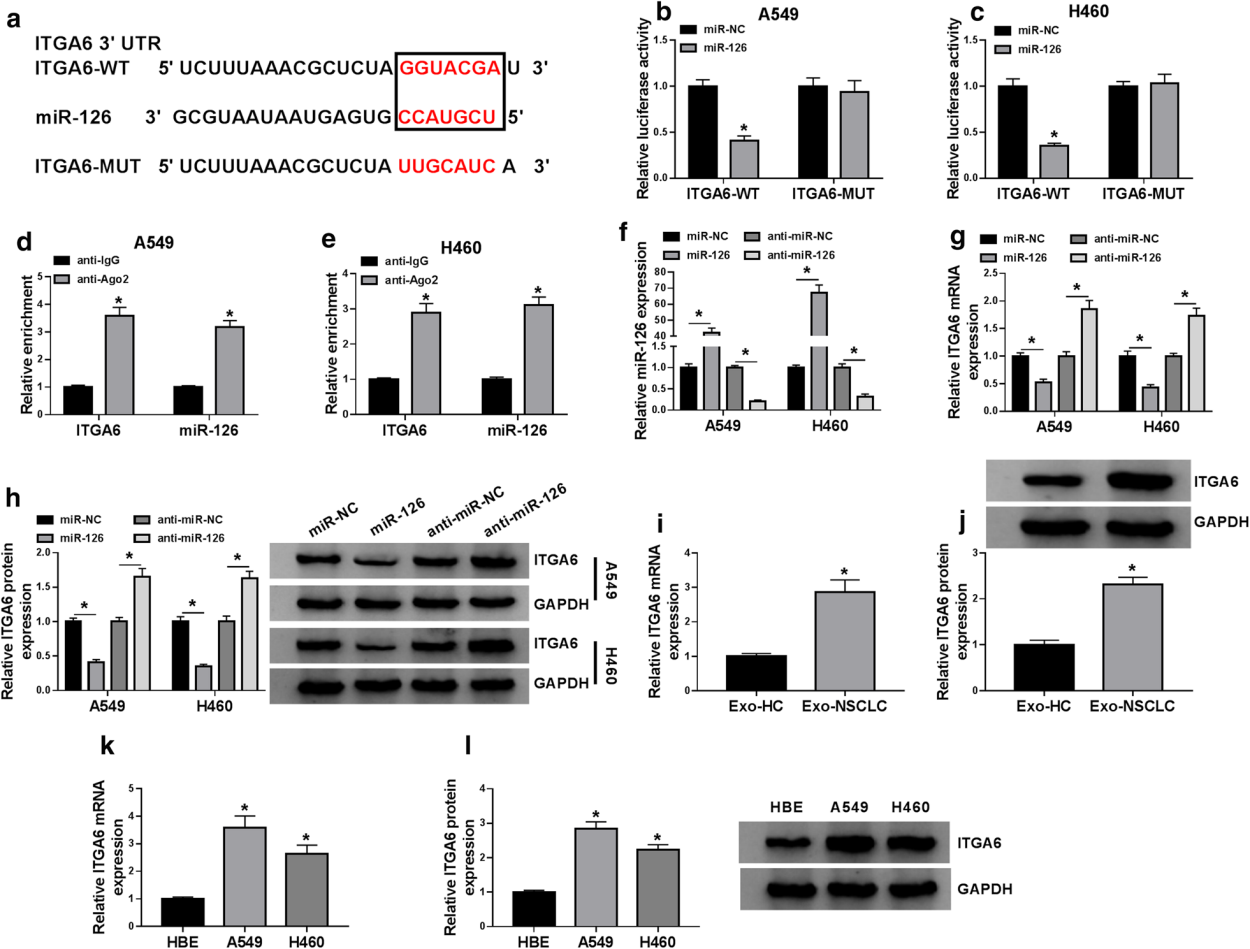
MiR-126 directly bound to ITGA6. **a** The binding site between miR-126 and ITGA6 3′UTR was analyzed by miRDB website. **b**, **c** The relationship between miR-126 and ITGA6 was validated by dual-luciferase reporter assay. **d**, **e** The relationship between miR-126 and ITGA6 was validated by RIP assay. **f** The efficiency of miR-126 mimic and miR-126 inhibitor was examined using qRT-PCR. **g**, **h** The effects of miR-126 mimic and miR-126 inhibitor on the expression of ITGA6 was determined by qRT-PCR and western blot. **i**, **j** The expression of ITGA6 in serum-derived exosomes from NSCLC patients and healthy controls was detected by qRT-PCR and western blot. **k**, **l** The expression of ITGA6 in HBE, A549 and H460 cells was detected by qRT-PCR and western blot. *P < 0.05

To explore the functional mechanism of miR-126, we investigated the potential targets of it. As shown in Fig. 4a, ITGA6 was one of the target mRNAs of miR-126, harboring a special binding site between its 3′UTR and miR-126 sequence. Then, the mutant sequence of ITGA6 3′UTR (mutation at miR-126 binding site) was generated. From dual-luciferase reporter assay, we discovered that the luciferase activity in A549 and H460 cells transfected with miR-126 and ITGA6-WT was notably decreased compared to miR-NC, while the luciferase activity in cells transfected with miR-126 and ITGA6-MUT was not changed compared to miR-NC (Fig. 4b, c). Besides, both ITGA6 and miR-126 could be richly detected in the anti-Ago2 group compared to the anti-IgG group in RIP assay (Fig. 4d, e). Next, the expression of miR-126 was notably promoted in A549 and H460 cells transfected with miR-126 compared to miR-NC but notably weakened in cells transfected with anti-miR-126 compared to anti-miR-NC (Fig. 4f). In miR-126-overexpressed cells, the expression of ITGA6 was impaired, while in miR-126-depleted cells, the expression of ITGA6 was reinforced at both mRNA and protein levels (Fig. 4g, h). Moreover, the expression of ITGA6 was higher in serum-derived exosomes from NSCLC patients than that from healthy controls (Fig. 4i, j), and the expression of ITGA6 was also elevated in A549 and H460 cells compared with that in HBE cells (Fig. 4k, l). The data suggested that ITGA6 was a target of miR-126.

### Exosomal MiR-126 restoration suppressed NSCLC malignant phenotypes by targeting ITGA6

**Fig. 5:**
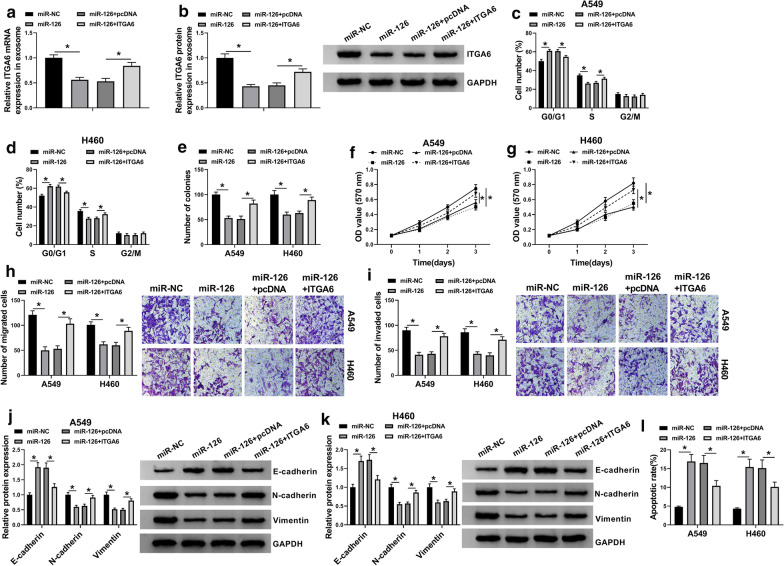
Exosomal miR-126 suppressed NSCLC cell malignant behaviors in vitro by targeting ITGA6. **a**, **b** The expression of ITGA6 in serum-derived exosomes transfected with miR-126, miR-NC, miR-126 + ITGA6 or miR-126 + pcDNA was determined by qRT-PCR and western blot. A549 and H460 cells were co-cultured with these transfected exosomes. In these cells, **c**, **d** cell cycle was investigated by flow cytometry assay. **e** The capacity of colony formation was assessed by colony formation assay. **f**, **g** Cell proliferation was assessed by MTT assay. **h**, **i** Cell migration and cell inmivasion were determined by transwell assay. **j**, **k** The expression of E-cadherin, N-cadherin and Vimentin was detected by western blot. **l** Cell apoptosis was monitored by flow cytometry assay. *P < 0.05

NSCLC serum-derived exosomes were transfected with miR-126 alone or miR-126 + ITGA6 together, with miR-NC or miR-126 + pcDNA as the control. The expression of ITGA6 weakened in exosomes transfected with miR-126 was largely recovered in exosomes transfected with miR-126 + ITGA6 at both mRNA and protein levels (Fig. 5a, b). Afterwards, A549 and H460 cells were co-cultured with these transfected exosomes. In function, exosomal miR-126-induced cell cycle arrest was notably alleviated by the reintroduction of ITGA6 in A549 and H460 cells (Fig. 5c, d). Besides, exosomal miR-126-blocked colony formation ability, cell proliferation, cell migration and invasion were also promoted by the overexpression of ITGA6 (Fig. 5e–i). The expression of E-cadherin was heightened, while the expression of N-cadherin and Vimentin was lessened in A549 and H460 cells containing miR-126-transfected exosomes, while the expression tendency of these markers was reversed in cells containing miR-126 + ITGA6-transfected exosomes (Fig. 5j, k). In addition, the stimulative apoptotic rate in A549 and H460 cells mixed with miR-126-transfected exosomes was partly inhibited in cells mixed with miR-126 + ITGA6-transfected exosomes (Fig. 5l). Therefore, exosomal miR-126 blocked NSCLC development in vitro by degrading ITGA6.

### Exosomal miR-126 inhibited tumor growth in vivo

**Fig. 6:**
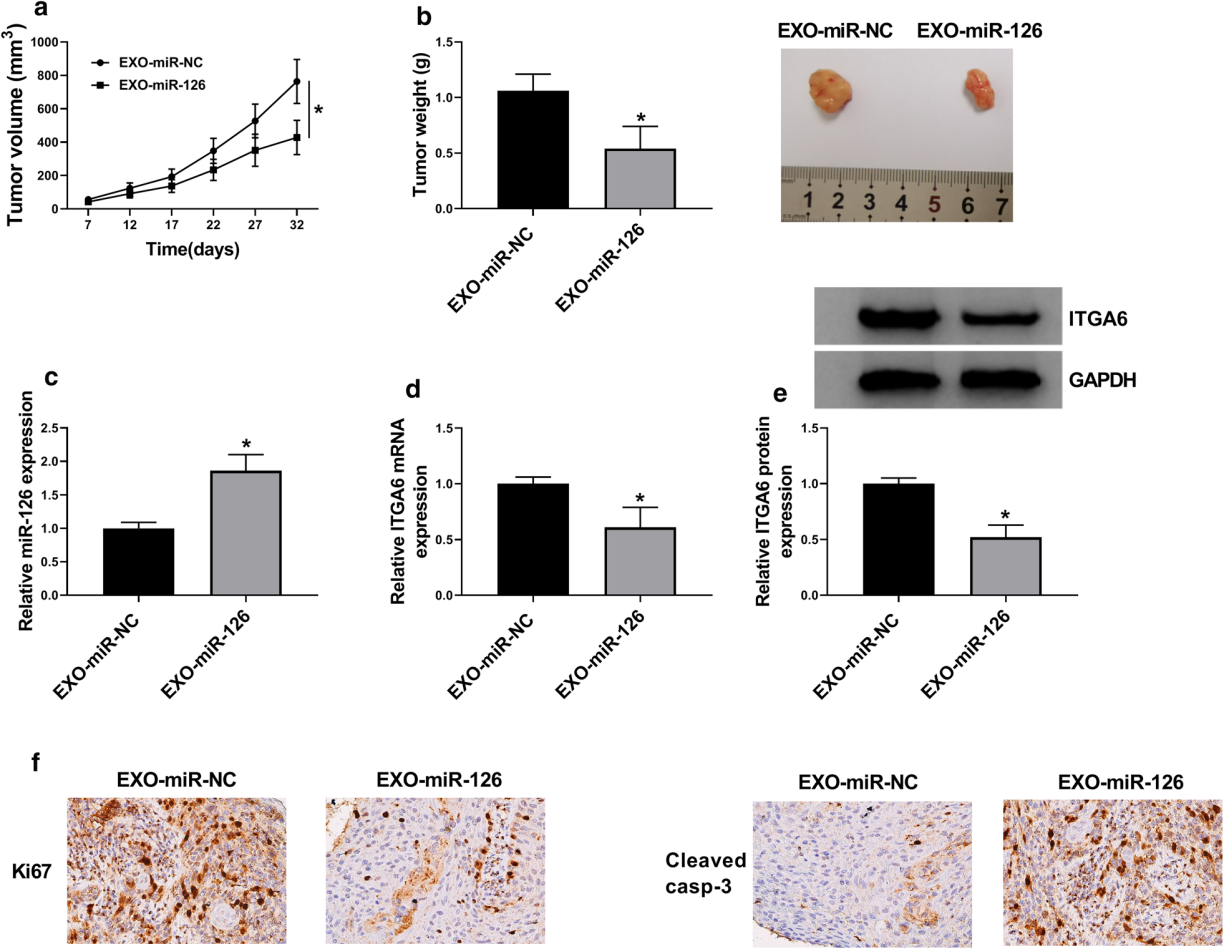
Exosomal miR-126 inhibited tumor growth in vivo. **a** Tumor volume was recorded in nude mice after EXO-miR-126 treatment. **b** Tumor weight was measured in nude mice after EXO-miR-126 treatment. **c** The expression of miR-126 in these tumor tissues was detected by qRT-PCR. **d**, **e** The expression of ITGA6 in these tumor tissues was detected by qRT-PCR and western blot. **f** IHC staining assay showed the expression of Ki67 and Cleaved casp3 in tumor tissues. *P < 0.05

Xenograft tumor models were established to determine the effect of exosomal miR-126 on tumor growth in vivo. As shown in Fig. 6a, b, exosomal miR-126 weakened tumor growth, including tumor volume and tumor weight compared to miR-NC. Moreover, the expression of miR-126 was elevated, while the expression of ITGA6 was lessened in mice treated with EXO-miR-126 compared with that treated with EXO-miR-NC (Fig. 6c–e). IHC staining assay presented that tumor tissues from the EXO-miR-126 group harbored higher expression level of Cleaved caspase-3 (Cleaved casp-3) and lower expression level of Ki67 compared with that in the EXO-miR-NC group (Fig. 6F). In short, exosomal miR-126 inhibited tumor growth in vivo by decreasing ITGA6 expression.

## Discussion

Cellular communication is essential for the development of cancers. Local tumor-stromal communication promotes microenvironment manipulation and optimizes tumor growth, invasion, and survival through inter-tumor communication [21]. It is increasingly clear that tumor-derived exosomes play an important role in intercellular communication by transporting various proteins, lipids and nucleic acids [22]. Numerous studies have shown that tumor-derived exosomes mediate the malignant development of tumor cells at least in part through the transport of miRNAs, and several exosomal miRNAs have been regarded as cancer biomarkers and therapeutic targets [23, 24]. Through our study, miR-126 was poorly expressed in serum-derived exosomes from NSCLC patients, and NSCLC serum-derived exosomes promoted NSCLC cell proliferation, migration/invasion and tumor growth. However, exosomes with enriched expression of miR-126 suppressed these malignant traits in NSCLC cells.

An important definition from a previous study was that exosomal miR-126 was a circulating biomarker in the development of NSCLC [18], and their data showed that exosomes containing enriched miR-126 from normal endothelial cells suppressed NSCLC cell proliferation [18]. Besides, miR-126 was reported to be downregulated in NSCLC tissues and cells, and low miR-126 expression was associated with tumor size and poor overall survival of NSCLC patients [25, 26]. In detail, miR-126 overexpression notably blocked NSCLC cell proliferation and solid tumor growth in nude mice [27]. These supporting data highlighted that miR-126 was a tumor suppressor at least in NSCLC. In our findings, NSCLC serum-derived exosomes harboring high expression of miR-126 induced NSCLC cell cycle arrest and apoptosis but weakened NSCLC cell proliferation, migration and invasion, suggesting that miR-126 could be transferred by exosomes and suppressed NSCLC development.

To figure out the mechanism of miR-126 action in NSCLC, we screened and identified the target mRNAs of miR-126. As a result, miR-126 could directly bind to ITGA6 3′UTR, which was further verified by dual-luciferase reporter assay. ITGA6 was shown to be upregulated in lung squamous cell carcinoma (LUSC) by GEPIA database (https://gepia.cancer-pku.cn/detail.php?gene=ITGA6). ITGA6 was a well-acknowledged oncogene in various cancers. For instance, ITGA6 synergistically interacted with RPSA to promote cell migration and invasion in pancreatic cancer [28]. ITGA6 was notarized to be a target of miR-143-3p, and miR-143-3p degraded the expression of ITGA6 to inhibit colorectal cancer metastases [29]. Moreover, high ITGA6 expression was involved in the occurrence and development of lung adenocarcinoma [30], and ITGA6 overexpression abolished the effects of miR-302a-5p to induced NSCLC cell proliferation and migration [31]. In our findings, consistently, ITGA6 expression was elevated in NSCLC serum-derived exosomes and cell lines. ITGA6 acted as a target of miR-126, and its overexpression reversed the inhibitory effects of miR-126 on NSCLC cell proliferation, migration and invasion.

In summary, we mainly evaluated the role of exosomal miR-126 in NSCLC and found that miR-126 overexpression in exosomes blocked NSCLC cell malignant traits and tumor growth in vivo, which was accomplished through the ITGA6-degraded mechanism. Exosomes-packaged miR-126 with high stability might be used as a new target for NSCLC diagnosis and treatment.

## Data Availability

The datasets used and/or analysed during the current study are available from the corresponding author on reasonable request.
