# Correction to: Exosomal miR-126 blocks the development of non-small cell lung cancer through the inhibition of ITGA6

**DOI:** 10.1186/s12935-021-01974-0

**Published:** 2021-06-02

**Authors:** Mingjun Li, Qianqian Wang, Xiaofei Zhang, Ningning Yan, Xingya Li

**Affiliations:** Department of Oncology, The First Affiliated Hospital of Zhengzhou University, No. 1 East Jianshe Road, Zhengzhou, 450052 Henan China

## Correction to: Cancer Cell Int (2020) 20:574 10.1186/s12935-020-01653-6

Following the publication of the original article [1], the authors notified us that one of the primer sequences in the article was wrong:

- Incorrect sequences: miR-126, F 5ʹ-TGTGGCTGTTAGGCATGG-3ʹ and R 5ʹ-AAGACTCAGGCCCAGGC-3ʹ
- Corrected sequences: miR-126, F 5ʹ-CGCGTCGTACCGTGAGTAAT-3ʹ and R 5ʹ-AGTGCAGGGTCCGAGGTATT-3ʹ

The original article has been corrected.
